# The state of targeted therapeutic pharmacological approaches in pediatric neurosurgery: report from the European Society for Pediatric Neurosurgery (ESPN) Consensus Conference 2024

**DOI:** 10.1007/s00381-025-06799-0

**Published:** 2025-04-02

**Authors:** P. Frassanito, U. W. Thomale, M. Obersnel, A. Romano, P. Leblond, F. Knerlich-Lukoschus, B. J. Due-Tønnessen, D. Thompson, F. Di Rocco

**Affiliations:** 1 Pediatric Neurosurgery, Fondazione Policlinico Universitario A. Gemelli IRCCS, Largo Agostino Gemelli, 8, 00168 Rome, Italy; 2 Pediatric Neurosurgery, Campus Virchow Klinikum, Charité Universitätsmedizin Berlin, Berlin, Germany; 3 Catholic University Medical School, Rome, Italy; 4 Pediatric Oncology, Fondazione Policlinico Universitario A. Gemelli IRCCS, Rome, Italy; 5 Department of Pediatric Oncology, Institute of Pediatric Hematology and Oncology, Leon Berard Comprehensive Cancer Center, Lyon, France; 6 Division of Pediatric Neurosurgery, Department of Neurosurgery, University Medical Center Göttingen, Göttingen, Germany; 7 Department of Neurosurgery, Oslo University Hospital - Rikshospitalet, Oslo, Norway; 8 Pediatric Neurosurgery, Great Ormond Street Hospital, London, UK; 9 Departement of Pediatric Neurosurgery, Hôpital Femme Mère Enfant, Lyon, France; 10 University of Medicine, Université Claude, Bernard 1, Lyon, France

**Keywords:** BRAF, Brain tumor, Druggable pathway, Glioma, MAPK, Personalized medicine, Targeted therapy

## Abstract

**Objective:**

The development of novel targeted therapies is opening new perspectives in the treatment of pediatric brain tumors. Their precise role in therapeutic protocols still needs still to be defined. Thus, these novel pharmacological approaches in pediatric neurosurgery were the topic of the European Society for Pediatric Neurosurgery (ESPN) Consensus Conference held in Lyon (France) in January 25–27, 2024.

**Method:**

The paper reviews the current knowledge about targeted therapy as well as the current literature published on the topic. The conference aimed for an interdisciplinary consensus debate among pediatric oncologists and pediatric neurosurgeons on the following questions.

- *Question 1: What is the current role for targeted therapies as neoadjuvant treatments before pediatric brain tumor removal?*
- *Question 2: What are the benefits, cost/efficiency, and long-term side effects of targeted therapies in the treatment of pediatric brain tumors?*
- *Question 3: Based on contemporary data, at which stage and in which pathologies do targeted therapies play a significant role?*

**Results:**

Ninety-two participants answered consensus polls on the state of the art of targeted therapies, the ethical issues related to their use, and the evolving change in the role of pediatric neurosurgeons. The neoadjuvant role of targeted therapies is difficult to define as there are many different entities to consider. Despite the recently reported potential benefits, questions regarding the use of targeted therapies are manifold, in particular regarding sustainable benefits and long-term side effects. Additionally, challenging cost issues is a limiting factor for the broader availability of these drugs. Studies have demonstrated superiority of targeted therapy compared to chemotherapy both in randomized trials and compared to historical cohorts in the management of a subset of low-grade gliomas. The same drug combinations, BRAFi and MEKi, may be effective in HGG that have relapsed, progressed, or failed to respond to first-line therapy. Similar conclusions on efficacy may be drawn for mTORi in TSC and selumetinib in plexiform neurofibromas. For other tumors, the picture is still obscure due to the lack of data or even the lack of suitable targets. In conclusion, targeted treatment may not always be the best option even when a target has been identified. Safe surgery remains to be a favorable option in the majority of cases.

**Conclusion:**

The constantly evolving drug technology and the absence of long-term safety and efficacy studies made it difficult to reach a consensus on the predefined questions. However, a report of the conference is summarizing the present debate and it might serve as a guideline for future perspectives and ongoing research.

## Introduction

Molecular advancements in pediatric brain tumors have revolutionized the classification of pediatric brain tumors [1]. Although the translation of these biological advancements into real clinical and therapeutical changes is yet to be realized, understanding the biology of a given tumor has helped to identify targetable alterations and relevant pathways and develop the so-called “targeted therapies” [2].

Several drugs are nowadays available and the number of clinical trials trying to define the role of these drugs is constantly increasing.

1. **Druggable pathways and available drugs**
  - **Mitogen-activated protein kinases (MAPK) pathways**
    Mitogen-activated protein kinase (MAPK) pathways transmit, amplify, and integrate signals by different extracellular stimuli and are involved in cell proliferation and differentiation [3].
    These transduction pathways consist of 3 to 5 layers of protein kinases, identified as MAP4K, MAP3K, MEK, MAPK, and MAPKAPK. Based on the components of the MAPK layer, 4 MAPK cascades have been defined: ERK1/2, JNK, p38 MAPK, and ERK5 [4].
    BRAF protein is a serine/threonine-protein kinase involved in the RAS-RAF-MEK-ERK signaling pathway that is an intracellular signal transducer activated by extracellular growth stimuli through specific transmembrane receptors. Activation starts with RAS-GTP binding to the RAS domain in RAF, leading to the activation of ARAF, BRAF, and CRAF proteins. This binding recruits RAF to the membrane and activates it via conformational changes. Consequently, RAF phosphorylates MEK and ERK, which activate downstream transcription factors like Elk-1, c-Fos, and c-Myc, influencing cell growth, differentiation, proliferation, and apoptosis [5, 6]. BRAF also activates MEK1/2, which subsequently activates ERK1/2. Growth factors such as epidermal growth factor (EGF) activate this pathway through receptor tyrosine kinases, enabling ERK to enter the nucleus and phosphorylate transcription factors [7].
    Aberrant signaling in the MAPK pathway can drive tumorigenesis because this signaling can lead to uncontrolled cell growth and survival [8]. Particularly, *BRAF* mutations have been found in various human tumors including CNS tumors (e.g., low- and high-grade pediatric glioma).
    Most *BRAF* mutations occur in the exon 11 and 15 kinase domains, disrupting stabilization of the kinase’s inactive form, leading to heightened BRAF activity and MAPK pathway activation [9]. *BRAF* alterations are classified into three classes. The prevalent *BRAF* mutations are class I mutations, which hyperactivate kinases and activate the MEK/ERK pathway independently of RAS activation or protein dimerization [10]; the most common class I mutation is V600E, which occurs due to a single-nucleotide substitution at position 1799T>A, resulting in replacement of valine (V) with glutamic acid (E) at codon 600. Class II alterations are rarer, including several exon point mutations (e.g., p.G464E/V, p.G469A/R/V, p.L597Q/V, p.K601E/N/T) and some fusion genes like the canonical *KIAA1549::BRAF* fusion, also independent of RAS, but require protein dimerization to activate MEK/ERK. Class III mutations exhibit low or no kinase activity and need upstream RAS activation and dimerization with CRAF for MEK/ERK activation [11, 12].
    RAF serves as a prime target for cancer drug development, particularly with first-class inhibitors like sorafenib, vemurafenib, and dabrafenib, which target mutated *BRAF* tumors. First-class BRAF inhibitors should not be used in tumors characterized by class II mutations because they may cause paradoxical upregulation of MAPK pathway signaling. To avoid the paradoxical activation of the MAPK pathway, type two RAF inhibitors (pan-RAF inhibitors) such as belvarafenib and tovorafenib have been developed and are currently under investigation in clinical trials [13, 14].
    MEK1/2 inhibitors currently available are selumetinib, trametinib, binimetinib, and cobimetinib. MEK1/2 inhibitors are useful for the treatment of *BRAF*-mutated tumors in combination with BRAF inhibitors, and as single agent for tumors harboring a *KIAA1549::BRAF* fusion not targetable by BRAFV600E inhibitors. By combining BRAF and MEK inhibitors, BRAF signaling can be attenuated, while the MEK inhibitor can suppress any mutant BRAF signaling not targeted by the target BRAF agent, thus inhibiting paradoxical activation resulting from the effect of BRAF inhibitors on BRAF dimers [15, 16].
    Fifteen to twenty percent of pediatric low-grade gliomas are characterized by the presence of the *BRAF V600E* mutation while the *KIAA1549::BRAF* fusion is observed in approximately one-third of cases. *BRAF V600E* has also been described in pediatric high-grade glioma but in a very low percentage of cases [14].
  - **PI3K/Akt/mTOR pathway**
    PI3K/Akt/mTOR Pathway regulates cell growth and proliferation. mTOR (mammalian target of rapamycin) responds to growth factors through the PI3K (phosphatidylinositol 3-kinase pathway). PI3K, activated by fibroblast growth factor receptors (FGFR) or insulin growth factor-1 receptors (IGF1-R) binding, causes the conversion of phosphatidylinositol-4,5-phosphate (PIP2) in the cell membrane into phosphatidylinositol3,4,5-phosphate (PIP3). PIP3 induces the phosphorylation and activation of AKT, which inhibits the complex tuberin–hamartin (TSC) which in turn disinhibits mTOR, promoting cell proliferation [17]. mTOR and MAPK are connected since mTOR is also activated by mitogenic signals transmitted via RAS/MEK/ERK [16].
    Alterations in the PI3K/Akt/mTOR pathway are frequent in different types of cancer, including pediatric high- and low-grade glioma [17].
    PI3K/AKT/mTOR pathway studies have led to the development of several distinct classes of drugs, including PI3K and AKT inhibitors, as well as allosteric mTOR and mTOR kinase inhibitors [18]. Everolimus, temosirolimus, and sirolimus are mTOR allosteric inhibitors; vistusertib is an mTOR kinase inhibitors; copanlisib, buparlisib, pilaralisib, paxalisib, alpelisib, and taselisib are PI3K inhibitors; ipatasertib is an AKT inhibitors [19, 20]. Among these target drugs, everolimus is the one for which there are studies in pediatric patients with glial tumors [21]. Paxalisib is being studied for the treatment of diffuse midline glioma [22].
  - **Receptor tyrosine kinase**
    Receptor tyrosine kinases (RTKs) are transmembrane receptors involved in signal transduction pathways that mediate cell-to-cell communication. The RTKs includes different families of receptor-like epidermal growth factor receptor (EGFR), platelet-derived growth factor receptors (PDGFR), fibroblast growth factor receptors (FGFRs), vascular endothelial growth factor receptors (VEGF), neurotrophic receptor kinase (NTRK) insulin-like growth factor receptors (IGFRs), and hepatocyte growth factor receptors (HGFRs/C-MET) [23].
    Growth factor ligands bind to extracellular regions of RTKs, and the receptor is activated by ligand-induced receptor dimerization and/or oligomerization with consequent autophosphorylation. Autophosphorylation of RTKs recruits and activates a wide variety of downstream signalling proteins including Ras/MAPK/ERK and PI3K/AKT, leading to cell proliferation, invasions, and angiogenesis [24]. Many RTKs have been implicated in the onset or progression of various cancers including pediatric high- and low-grade gliomas. Numerous studies have highlighted RTKs mutations in pediatric high-grade glioma (pHGG). Approximately 20–30% of patients with pHGG have mutations and/or amplifications of platelet-derived growth factor receptor alpha (PDGFRA) while 10% have MET fusions and 3–7% MET amplification. Other RTK gene fusions involve the ALK, ROS1, FGFR, MET, and NTRK genes [25].
    Several case reports have been published demonstrating the efficacy of targeting ROS1, FGFR, NTRK, and MET gene fusions in pHGG [26, 27]. Larotrectinib, a highly selective TRK inhibitor, demonstrated rapid and durable responses, high disease control rate, and a favorable safety profile in patients with TRK fusion-positive CNS tumors [28]. Other NTRK inhibitors such as entrectinib and reprotrectinib are currently under investigations in patients with tumors harboring *NTRK* or *ROS1* alterations.
  - **Epigenetic alterations**
    Epigenetic alterations refer to modifications in gene expression and include alterations in DNA methylation, histone methylation/acetylation, chromatin remodeling, and regulation of non-coding RNA. These alterations contribute to tumor aggressiveness by dysregulating key developmental pathways, genomic stability, and cell cycle control [29]. Consequently, genes responsible for histone modifiers are crucial epigenetic regulators; their dysregulation can enhance tumorigenesis and resistance to therapies [30].
    The sequencing of pHGGs revealed significant genetic differences from adult gliomas, with a higher frequency of mutations in epigenetic key drivers. These mutations, particularly in histones H3.1/H3.3 and chromatin remodelers like ATRX, DAXX, and SETD2, suggest epigenetic disruption of neural cells as a key factor in gliomagenesis [29]. Drugs acting on epigenetic alterations, like histone deacetylase inhibitors (HDACs) or EZH2 inhibitors, could represent a promising treatment for pHGGs.
    Acetylation and methylation of histones play a crucial role in the epigenetic regulation of gene expression by modifying the structure of chromatin and modulating the access of transcription factors to the target DNA. These modifications are particularly enriched in transcriptionally active regions, such as promoters and enhancers. Acetylation relaxes the bonds between protein cores and DNA, allowing transcription factors and bromodomain proteins to bind more easily. The acetylation state is regulated by histone acetyltransferases (HATs) and histone deacetylases (HDACs) [31]. HDAC inhibitors are a class of drugs acting on this process of DNA regulation with consequent cancer proliferation arrest.
    Enhancer of zeste homolog 2 (EZH2) is the catalytic subunit of polycomb repressive complex 2 (PRC2) that modifies gene expression via H3K27 trimethylation. EZH2 also influences other regulatory mechanisms and plays significant roles in cell proliferation, apoptosis, senescence, and cancer pathophysiology, making it a vital therapeutic target [32]. EZH2 inhibitors could represent good prospects for the treatment of brain tumors.
  - **VEGF**
    High-grade gliomas are characterized by rapid growth associated with angiogenesis. Numerous studies have demonstrated that inhibition of VEGF expression is able to reduce the formation of blood vessels and consequently tumor growth. Bevacizumab, a recombinant humanized monoclonal antibody, binds all isoforms of VEGF with high affinity and specificity [33]. Bevacizumab showed effectiveness in improving progression-free survival when used in combination with other drugs [34]. Unfortunately, adding bevacizumab to the combination radiotherapy-temozolomide did not improve EFS in children with newly diagnosed high-grade glioma [35]. Also, bevacizumab showed clinical efficacy in pediatric low-grade gliomas and significant visual improvement in optic pathway gliomas, with a favorable toxicity profile [8, 36, 37].
  - **Sonic Hedgehog pathway**
    The Sonic Hedgehog (Shh) signaling pathway is a crucial network that regulates key events during developmental processes, such as growth and the formation of multicellular embryos [38]. Alterations in the regulation and transduction of the Shh pathway are associated with birth defects, tissue regeneration, stem cell renewal, and tumor growth. The activation of Shh signaling requires the binding of Shh to the PTCH1-Smo receptor complex. This heterodimeric complex consists of the transmembrane subunits PATCH1 and Smo. The binding of Shh to PATCH1 activates and stabilizes Smo, which initiates a signaling cascade through G protein-coupled receptor-like proteins, affecting transcription factors such as Ci and Gli which regulate the expression of gene involved in cell survival, proliferation, and differentiation [39]. Mutations in this pathway cause the initiation and progression of the SHH medulloblastoma subtype and could be a target of possible treatments: indeed, there are phase I and phase II clinical trials that investigate the role of vismodegib and sonidegib, with promising results [40].

2. **Available studies**
  - ***Low-grade glioma***
    Pediatric low-grade glioma (LGG), representing 30–40% of all CNS tumors in children, is now commonly accepted as a chronic disease, so the aim of treatment is focused on functional outcomes, minimizing long-term morbidities to maximize quality of life [41].
    These tumors are mainly characterized by aberrant intracellular signaling via the MAPK pathway, so targeted therapies against *BRAF* alterations have been studied.
    In tumors with *BRAF V600E* mutation, dabrafenib was investigated in a phase I/IIa study, with promising results (32 patients enrolled, minimum follow-up 26.2 months, overall response rate 44%) [42]. Vemurafenib was also studied in a phase I study and demonstrated promising anti-tumor activity in recurrent tumors with manageable toxicity, and allowing the start of an ongoing phase 2 study [43]. In 2023, Bouffet et al. demonstrated in a phase 2 trial that a combination of dabrafenib and trametinib shows a better tumor response rate (47%) and progression-free survival (20.1 months) compared to chemotherapy (11% and 7.4 months respectively), with a lower rate of adverse events [44]. In 2023, the FDA approved this drug combination for pLGG with *BRAF V600E* mutation as a first-line treatment [45].
    In case of recurrent and progressive pLGG associated with neurofibromatosis type 1 or with a *KIAA1549::BRAF* fusion, a phase II trial using the MEK inhibitor selumetinib demonstrated significant rates of partial responses (30–40%) and diseases stabilization (50–60%) [46–48].
    The second-generation pan-RAF inhibitor (tovorafenib) has been investigated in the multicentric FIREFLY-1 phase 2 study, as a monotherapy in BRAF-altered relapsed/refractory tumors, obtaining a 64% overall response among 69 children. Interestingly, this trial demonstrated significant responses in patients previously treated with BRAF or MEK inhibitors [13]. The LOGGIC-FIREFLY2 phase III study comparing single-agent tovorafenib and chemotherapy is currently ongoing.
    A phase II trial (POETIC study) provided a possible treatment of radiographically progressive pLGG using everolimus. Among 23 evaluable patients, 2 had a partial response, 10 had stable disease, and 11 had clinical or radiographic progression [21].
    Bevacizumab has evidence of effectiveness mostly in optic pathway glioma [8, 49, 50]. A recent retrospective study collected 88 children, showing good visual outcomes (29% improvement, 49% stabilization) and radiographic outcomes (40% partial response, 49% stability, 11% progression), as a monotherapy or in association with irinotecan [36].
    Although *IDH1* or *IDH2* mutant low-grade diffuse glioma are very rare in children, the double-blind phase III trial INDIGO deserves mention. This trial involving 331 patients without prior treatment other than surgery, demonstrated the efficacy of vorasidenib, an oral brain-penetrant inhibitor of IDH1 and IDH2 enzymes. Comparing the vorasidenib group with the placebo group, the trial showed significantly improved progression-free survival (Hazard ratio 0.39) and time to next intervention (Hazard ratio 0.26), with a predominantly low-grade safety profile [51].
  - ***High-grade glioma***
    In adults, targeted therapies have so far failed to improve the prognosis of high-grade gliomas (HGG). An important tumor heterogeneity is described in HGG, but the targetable alterations are mostly passenger mutations, that do not contribute to cancer development. On the other side, the most frequent driver mutations (TERT promoter mutation), that contribute to oncogenesis, are not yet targetable. Also, blood-brain barrier remains an issue in rare glioblastoma with targetable alterations (e.g., FGFR fusions < 3% cases) [52].
    The standard treatment for pediatric patients with HGG remains surgery followed by radiotherapy and temozolomide, as also suggested for adult patients in the well-known Stupp’s regimen [53]. In HGG, complete resectional surgery is often impossible or not recommended, and radiotherapy to a large volume in very young children can have unacceptable short- and long-term side effects [54].
    Rearrangements in *ALK*, *ROS1* and *NTRK* genes result in fusion proteins that are oncogenic drivers of many pediatric tumors, including pediatric HGG [55]. Entrectinib and larotrectinib, inhibitors of TRKA/B/C, ROS1, and ALK, have already been approved for solid tumors with these alterations. There are promising ongoing phase I and II trials, showing that these drugs have a rapid and durable activity over medium-term follow-up, with an acceptable rate of adverse effects [56].
    Approximately 5 to 10% of pediatric HGG are driven by somatic MAPK pathway alterations, most commonly by somatic point mutation in the *BRAF* oncogene (the most common is V600E). The use of a combination of BRAF and MEK inhibitors, already studied in phase II clinical trials in LGG with good results [57], is debated in the context of HGG. There is an ongoing phase II trial combining dabrafenib and trametinib but results of this are pending (NCT02684058). A retrospective study, albeit a small sample of 19 patients with HGG treated with a BRAF inhibitor with or without the additional MEK inhibitor, showed superior clinical outcomes compared to historical data [57].
    EGFR, FGRF, and MET overexpression in glioma is associated with poor prognosis and greater tumor invasion [58]; however, these may serve as potential therapeutic targets. Ongoing phase II trial are investigating the use of drugs such as nimotuzumab [59], erlotinib [60] (EGFR inhibitors), also in combinations with mTOR/PI3K inhibitors, erdafitinib (FGFR inhibitors) in such tumors. MET inhibitors, as volitinib, are currently under a phase I trial investigation [58].
    A PARP inhibitor, olaparib, is currently being investigated in a phase II trial against HGG [61].
    Very rare cases of pHGG with constitutional mismatch repair deficiency (CMMRD) were also treated with anti-PD1 as nivolumab, which reportedly contributed to prolonged survival [62].
    Finally, larotrectinib, a highly selective TRK inhibitor, demonstrated good outcomes in *NTRK*-fused low-grade and high-grade gliomas, with a global progression-free survival of 56% and an overall survival of 85% at 12 months in 33 patients evaluated [28].
  - ***Diffuse midline glioma***
    Diffuse midline glioma H3K27-altered has a poor prognosis, with a median overall survival of 9–12 months post-diagnosis, despite the current therapies. Complete resection is impossible, due to the localization and infiltration of the tumor, but stereotactic biopsy is often recommended to better define tumour biology prior to enrolment in clinical trials investigating new targeted therapies.
    There are promising expectations with targeted therapy, with clinical trials in phase I and several preclinical studies [63].
    In particular, there are preclinical studies with histone deacetylase and demethylase inhibitors counter H3K27M mutation, agents against ACVR1 receptor (present in circa 32% of H3K27M-mutant DMG), ALK2 inhibitors, MEK1/2 inhibitors, EZH2 inhibition (PRC2), and PI3K/mTOR inhibitor [58].
    Phase II trials are currently investigating ONC201 (imipridone) in pediatric H3K27M-positive gliomas. ONC201 is an antagonist of the dopamine receptors D2/3 and an activator of the mitochondrial caseinolytic protease P (CIpP), resulting in upregulation of the pro-apoptotic TRAIL receptor, which induces cancer cell death. First data emerging from trials demonstrate a good tolerability of the drug and promising results, with a longer progression-free period and finding of radiographic regressions [64–66]. The BIOMEDE 2.0 study (NCT05476939) is a multicenter, randomized, open-label, controlled phase-3 trial evaluating efficacy of ONC201 in comparison with everolimus in combination with radiation therapy.
    Metabolic inhibitors, targeting polyamine synthesis and polyamine transport, or shifting the glucose metabolism to mitochondrial-dependent oxidative phosphorylation, are also promising factors in preclinical studies [67–69].
    CAR T-cell therapy directed against GD2, a tumor-associated cell surface antigen, seems effective in preclinical studies [70]. Furthermore TILs (tumor-infiltrating lymphocytes), already used in trials for other tumors and well tolerated, look promising, although a larger volume of tumor tissue would be needed for this approach, rather than the little obtained by a stereotactic biopsy [71].
    An ongoing phase Ib trial uses autologous dendritic cells pulsed with an allogeneic tumor cell-line lysate to reactivate tumor-specific T cells, after irradiation, that generate a DIMG-specific immune response detected in peripheral blood mononuclear cells and CSF [72]. Also, the immune-modulating antibody MDV9300 (pidilizumab) is a potentially promising treatment after radiotherapy: of the nine pediatric patients enrolled in the study, two were still alive nearly 30 months from diagnosis at the trial conclusion, with radiographically defined disease stability [73].
    Finally, DNX-2401, a replication competent, genetically modified virus that stimulates an anti-tumor immune response, is under investigation in an ongoing phase I clinical trial (NCT03178032) [74].
    There are clinical trials of intratumoral drug delivery via convection-enhanced delivery (CED), with IL13-Pseudomonas exotoxin [75], obtaining a partial response, or monoclonal antibodies targeting glioma-associated antigen conjugated to a radioisotope, with no systemic toxicity and a little increment of overall survival [76]. Another ongoing phase I study is investigating irinotecan liposome injection using real-time imaging with gadolinium (NCT03086616).
    Finally, a phase I trial uses a super-selective intraarterial cerebral infusion of bevacizumab and cetuximab, using mannitol or magnetic resonance-guided focused ultrasound to temporarily open the blood-brain barrier, with a little increment of overall survival and some radiological response [77].
  - ***Medulloblastoma***
    Paradoxically, the molecular landscape of medulloblastoma is now well established [78]; however, targeted therapies have been investigated only in the very restricted subgroup of SHH-medulloblastomas.
    Smoothened inhibitors (SMO-i) recently entered clinical trials for Sonic Hedgehog-driven medulloblastoma, but with a highly variable clinical response [79].
    Genome sequencing of SHH medulloblastoma may predict genotype-related response to SMO-I, contraindicating the use of this drug in SUFU-mutated high-risk SHH medulloblastoma [79].
    Phase I trials have confirmed the tolerability of vismodegib and sonidegib [80, 81], and phase II trials showed an increased progression-free survival only in patients with SHH mutation [82].
    This treatment is not used as first-line therapy in young children. Open questions remain about the possibility of combining targeted therapy with conventional chemotherapy, as well as the possibility to consider topical administration of these drugs or via intraventricular access device.
    Further studies are required to find druggable targets and possible therapies in medulloblastoma, also in other molecular subgroups.
  - ***Craniopharyngioma***
    Current therapeutic strategies for both adamantinomatous (ACP) and papillary craniopharyngiomas (PCP) include surgery, often followed by adjuvant radiotherapy in case of a subtotal resection or at relapse. Several molecular mechanisms involved in CP pathophysiology have been discovered, and these molecular aberrations might be considered for targeted therapy [83].
    PCP has the most encouraging results, using BRAF inhibitors in *BRAF V600E* mutated PCP; however, this pathology is less common in children compared with adults.
    ACP is a more relevant pathology in the pediatric age group. Numerous molecular pathways have been described [84], and in most cases, *CTNNB1* mutations activating the Wnt pathway can be recognized. There are ongoing early phase clinical trials, which are investigating the effectors of Wnt pathway inhibition in adult patients with advanced solid tumors. A clinical trial of the combination of anti-PD1 nivolumab with the pan-RAF tovorafenib is currently ongoing in pediatric CP (NCT05465174).
    The involvement of the MAPK/ERK pathway in ACP pathogenesis was also described, so MEK inhibitors, such as binimetinib, was used in a single case, a multi-treated 26-year-old female, with a remarkable decrease in tumor size at 8-month follow-up.
    Interferon (IFN) is the most extensively studied form of immunotherapy. Intracystic IFNα proved to be safe and effective, as part of multimodal management of adamnatimomatous CRF, although this drug is no longer available and has been replaced by its pegylated form [85]. A clinical study involving 15 children aged ≤ 21 showed a demonstrable radiological response in 3 cases, but only 1 patient showed also a parallel clinical response. Another study, involving 5 children, was conducted using pegylated interferon alfa-2b: results were promising, as 2 children had a complete response, 2 a partial response and 1 a stable disease. Based on this study, another was conducted using peginterferon alfa-2b, but only 2 of the 18 patients had a partial response, and only 1 had a radiological improvement for more than 3 months [86].
    Immune system-targeted therapy is another potential option. Tocilizumab (an antibody against IL-6) was offered in two children, on a compassionate basis, after failure of conventional treatment. A radiological reduction in size at 6 months follow-up was observed. The combination therapy with tocilizumab and bevacizumab showed a significant radiological reduction in cysts’ size [87]. At the moment, the efficacy of tocilizumab is being studied in a phase 0 open-label clinical study (NCT03970226).
  - ***SEGA***
    Subependymal giant cell astrocytomas (SEGA), presenting almost exclusively in tuberous sclerosis complex (mutations *TSC1*, *TSC2*), are generally benign, slow-growing, non-infiltrative lesions, although they may be more aggressive from a clinical standpoint [88]. Surgical resection is the first-line treatment, when the tumor is symptomatic. From 2010, a medical therapy with mTOR inhibitors, namely sirolimus and everolimus, the latter showing more favorable pharmacokinetic characteristics, has been approved.
    Medical therapy is primarily recommended in instances of asymptomatic tumor progression, when surgery is not indicated, in case of systemic contraindications, or in the case of recurrent tumors, as well as multiple tumors, which are often bilateral. These drugs can also be used as neoadjuvant treatment in tumors infiltrating deep structures, to reduce tumor size to facilitate safer surgery. Finally, mTORi are used as adjuvant therapy in case of subtotal resection [89].
  - ***Plexiform neurofibroma***
    Plexiform neurofibromas are benign tumors, but locally aggressive and with a 10% risk of transformation into malignant peripheral nerve sheath tumors (MPNSTs) [90].
    Selumetinib has been approved for treatment of progressive inoperable and symptomatic plexiform neurofibromas in pediatric patients >3 years old with neurofibromatosis type 1. This is a highly selective MEK 1/2 inhibitor, investigated in the SPRINT Clinical Trial, that shows volume reduction in 68% of 74 treated patients, with durable responses (more than 1 year), with a small number of patients showing a slow progressive disease and with acceptable toxic effects in most cases [91].

3. **Side effects and costs**
  Cost is a prominent issue limiting the use of targeted therapies. As an example, dabrafenib and trametinb regimen costs more than 40,000 euros per year per patient. Similarly, ONC201 regimen costs more than 50,000 euros per year. The main goal to reduce the cost is to have these drugs approved by continental medicine agencies (FDA and EMA).
  In this context, drug repurposing should be considered as an alternative [92–94]. This approach is faster, cheaper, and more accessible than the development of new targeted drugs. It increases the number of drugs that can be used and may be effective on multiple targets. Additional advantages include lower toxicity profiles of drugs already in use and their potential to be combined with other treatment options. As an example, a protocol against HGG using hydroxychloroquine, perampanel, mebendazole, and metformin was recently presented *(oral communication, SIOPe Brain Tumor Group meeting in Philadelphia, USA, on June 27, 2024)*.
  Nonetheless, targeted therapies are not free from complications and side effects, with skin toxicity being the most common [95].
  BRAF and MEK inhibitors show overlap in their toxicity, which is anticipated as these target the same pathway. Dermatological adverse events occur in up to 60% and include maculopapular rash, dry skin, photosensitivity, acne, and alopecia. Interestingly, there are case reports of intolerance of the adverse side effects of one BRAFi, which appear to be neutralized when combined with another agent which has a more acceptable side effect profile [95]. For example, dabrafenib/trametinib when used as combination therapy are better tolerated than monotherapy with MEKi.
  Other less common adverse events of these agents include hepatotoxicity, pyrexia (occurs more commonly when BRAFi combined with MEKi), QT prolongation, weight gain, hypertension, pericarditis, uveitis, arthralgias, fatigue, vomiting, diarrhea, and/or mucositis. Thus, patients require close surveillance with regular skin exams, ophthalmologic assessment, evaluation of liver function, and cardiac assessment.
  Similarly, panRAFi are relatively well tolerated with overlapping toxicity profile compared to MEKi and BRAFi, with skin toxicity (specifically maculopapular rash) being the most common AE. Notably, there were no reported ocular toxicity, cardiac toxicity, or weight gain with tovorafenib. Other side effects include hair color changes, elevated CPK, and anemia. A significant decrease in growth velocity has been reported with tovorafenib, without associated bone age advancement or premature fusion of growth plates (also reported with SMO inhibitors vismodegib and sonidegib). Indeed, growth velocity recovers after drug discontinuation [96].
  Skin rashes are also common with other drugs, such as EGFRi (e.g., erlotinib) and PDGFRAi (e.g., dasatinib), while melanodermia is a potential side effect of VEGFi (bevacizumab) and nails problem with FGRFi (e.g., erdafinitib) or paronychia with MEKi (e.g., trametinib). Changes of color hair have been also reported as a class effect with long duration of TRKi.
  In general, these adverse events are reversible when targeted therapy is stopped.
  Endocrinological complications are frequent with anti-PD1 drugs as nivolumab [97], with a higher risk of autoimmune syndromes, but less common with other drugs. FGFR1i may cause bone density loss, C-kit inhibitors growth impairment, and SHHi cartilage closure. Other drugs may impair the hormonal status, in particular immunotherapy (PD1i) may cause hypopituitarysm, VEGFi hypothyroidism, and cabozantinib hypogonadism.
  Autoimmune multiorgan toxicities have been also reported [98]
  Toxicity, either acute or chronic, is therefore one of the commonest reasons to stop targeted therapies. Knowledge of the pharmacological profile of these drugs is relevant for the neurosurgeon, not only for the identification of toxicity but also in recognition of the need for an interval between stopping therapy before proceeding to surgery (Table 1). For example, bevacizumab should be discontinued at least 3 weeks before surgery to reduce the bleeding risk and healing issues. In emergency setting, plasma exchange has been used [99].

**Table 1:** Side effects of most common targeted therapies

*Drug*	*Brand name*	*Molecular action*	*Indication*	*Side effects*	*When to stop*	*Other risks*
Nivolumab	Opdivo	PD1i	Used in DIPG and in tumor with tumor mutation burden ≥ 5 mutations/megabase (mut/Mb) and/or mismatch repair deficiency	Constipation, weight loss, dry mouth and/or mouth sores, flu-like signs	Discontinued 4–5 days before surgery	Rare autoimmune adverse event
Dabrafenib	Tafinlar	BRAFi	BRAFV600E	Altered blood counts, skin changes	Discontinued 1 day before surgery	Rebound when stopped
Bevacizumab	Avastin	VEGFi	Used in low- and high-grade gliomas	High blood pressure, proteinuria, bleeding, headache, dry skin	Discontinued > 20 days before surgery *(emergency: plasma exchange?)*	Risk of bleeding, wound breakdown (start > 28 days after surgery)
Trametinib	Mekinist	MEKi	KIAA1549-BRAF fusion, NF1	Acne, perionyxis	Discontinued 5 days before surgery	Rebound when stopped
Everolimus	Votubia, Afinitor	mTORi	TSC	Stomatitis, acne, “radiation recall syndrome”	Discontinued 7–14 days before surgery	Problems with wound healing, avoid concomitant ketogenic diet
  The effects of long treatment exposure to targeted agents, including neurodevelopment, are unknown. This aspect is particularly important when dealing with benign tumors and a young population. Similarly, little is known about rebound on stopping treatment or addiction, as well as the risk of developing resistance.
  An example is given by a case of unexpected and unprecedented acceleration of tumor growth of optic pathway glioma after sorafenib administration. A weak BRAF inhibition resulted in a paradoxical activation (retrocontrol) of the pathway [100].
  Targeted therapy should usually be stopped during the bridge to other treatments, although the best combination of treatments is far to be defined.

Questions and dilemmas, however, remain to define the optimal relationship between different treatment modalities, such as surgery, radiation therapy, and chemotherapy. An ongoing debate supported by a stronger evidence base will be necessary among the disciplines in order to better understand the new potentials and to select balanced protocols for future treatments of pediatric brain tumors. For these reasons, the European Society for Pediatric Neurosurgery (ESPN) promoted an international consensus meeting hosted and initiated by author F.D.R. to review current evidence together with its potentials and drawbacks, underpinning the current and future role of targeted therapies in pediatric brain tumors. The aim was to review currently published research on targeted therapies and to elaborate a consensus on their use when managing brain tumors within the context of a pediatric multidisciplinary neuro-oncology team.

## Methods

A Consensus Conference under the auspices of the European Society of Pediatric Neurosurgery (ESPN) was held in Lyon, France, on 25–27 January 2024 (CPN2024). The 92 participants comprised pediatric neurosurgeons, neuroncologists, pathologists, and neurologists who reviewed evidence relating to the current role of targeted therapies in the management of pediatric brain tumors. The stated aims were to review the state of the art in targeted therapies and to attempt to seek consensus, if possible, with the intention to answer to the following questions:

- *Question 1: What is the current role for targeted therapies as neoadjuvant treatments before pediatric brain tumor removal?*
- *Question 2: What are the benefits, cost/efficiency, and long-term side effects of targeted therapies in the treatment of pediatric brain tumors?*
- *Question 3: Based on contemporary data, at which stage and in which pathologies do targeted therapies play a significant role?*

The questions were meant to serve as guidelines to lead through the entire meeting. Although these questions are difficult to answer due to the constantly evolving scenario and the absence of long-term data, these will thus be further elucidated in the “Discussion” section.

In addition, consensus polls on the state of the art of targeted therapies were performed which addressed common sense of treatment strategies, ethical issues, and evolving role of pediatric neurosurgeon in the context of newly developed targeted therapies.

## Results

Answers to the poll are summarized in Table 2 and discussed in the following sections.

**Table 2:** Consensus poll

Questions	Pre-meeting		Post-meeting	
	No (%)	Yes (%)	No (%)	Yes (%)
*State of the art of targeted therapies*				
Do you consider that targeted therapy will reduce the number of brain tumor surgery in the future?	**39.47**	**60.53**	**45.45**	**54.55**
Do you consider that targeted therapy will improve the clinical outcome of brain tumor patients?	**2.63**	**97.37**	**5.45**	**94.55**
Should neurosurgeons always be involved in decision-making in the field of targeted therapies in neuro-oncology?	**2.63**	**97.37**	**3.64**	**96.36**
Should neurosurgeons be involved in the prescription of targeted therapies in neuro-oncology?			**50.0**	**50.0**
*Ethical questions in pediatric neuro-oncology*				
It is acceptable to offer a family a randomization between surgery versus medical treatment?			**39.58**	**60.42**
Should the decision between neoadjuvant versus adjuvant (post-surgical) medical therapy better be regulated by protocol?			**8.0**	**92.0**
*Future perspectives*				
Should neurosurgeons always contribute to the design of novel oncological treatment protocols?			**6.0**	**94.0**
Do we need more multicenter prospective randomized trials on open neurosurgical questions?			**8.0**	**92.0**
The possibility of bias related to center and surgeon expertise represents a contradiction to perform a prospective randomized multicenter surgical trial?			**35.42**	**64.58**

### Consensus poll: State of the art of targeted therapies

1. *Do you consider that targeted therapy will reduce the number of brain tumor surgery in the future (NO/YES)?*

*Pre-meeting 39.47%/60.53%*

*Post-meeting 45.45/54.55*

2. *Do you consider that targeted therapy will improve the clinical outcome of brain tumor patients (NO/YES)?*

*Pre-meeting 2.63/97.37*

*Post-meeting 5.45/94.55*

3. *Should neurosurgeons always be involved in decision-making in the field of targeted therapies in neuro-oncology (NO/YES)?* 

*Pre-meeting 2.63/97.37*

*Post-meeting 3.64/96.36*

4. *Should neurosurgeons be involved in the prescription of targeted therapies in neuro-oncology (NO/YES)?*

*50.0/50.0*

***Comment:*** *Almost two thirds of participants anticipated that targeted therapies would reduce the future need for conventional therapies, including brain tumor surgery; this proportion reduced somewhat after informed discussion. Participants were unable to speculate on the number of brain surgeries that will be required in the era of targeted therapies; however, there was agreement that neurosurgeons will more frequently be asked to perform biopsies to better understand the molecular basis of individual tumours both at the time of presentation and at recurrence.*

*There was almost unanimous agreement that targeted therapies will improve the outcome of brain tumor patients.*

*Although participants agreed that neurosurgeons should be involved in the treatment decision-making, it was more controversial whether neurosurgeons should be also involved in the prescription of targeted therapies. This may reflect the composition of the participants in the meeting, in terms of neurosurgeons claiming a more active involvement and neuro-oncologists preserving their role.*

### Consensus poll: Ethical issues

1. *It is acceptable to offer a family a randomization between surgery versus medical treatment (NO/YES)?*

*39.58%/60.42%*

2. *Should the decision between neoadjuvant versus adjuvant (post-surgical) medical therapy better be regulated by protocol (NO/YES)?*

*8.0/92.0*

***Comment:**** Randomization between surgery versus medical treatment was not acceptable by one third of the participants. This may reflect the larger proportion of neurosurgical participants as well as the inherent limitations of surgical trials. Surgical trials are difficult to set up and to conclude, as only half of the initiated trials reach their recruitment target. Additional challenges of surgical trials include the problem of offering radically different treatment choices to patients and families, for example, randomization between an operation and no operation. Patients or clinicians often have *a priori* preferences for one or the other treatment, which may be further compromised by imbalanced presentation of the treatment options to patients. Inherent variations in surgical experience, case volume, and difficulties with randomization in emergency situations were also identified as problems in surgical trials.*

*Although randomized-controlled trials remain the gold standard for generating evidence on what is the best treatment for a given condition or in a specific setting, observational studies have some advantages when compared to RCTs, such as lower cost, greater timeliness, and a broader range of patients eligible for study inclusion, thus providing, in some cases, quality evidence comparable to RCTs. The level of evidence gained from a poor-quality RCT is not necessarily better than that from a well-conducted cohort stud*y [101].

*Regardless of the type of study, the participants almost unanimously agree that well established protocols should regulate the decision on the use of neoadjuvant versus adjuvant medical therapy.*

### Consensus poll: Future perspectives

1. *Should neurosurgeons always contribute to the design of novel oncological treatment protocols (NO/YES)?*

*6.0%/94.0%*

2. *Do we need more multicenter prospective randomized trials on open neurosurgical questions (NO/YES)?*

*8.0/92.0*

3. *The possibility of bias related to center and surgeon expertise represents a contradiction to perform a prospective randomized multicenter surgical trial (NO/YES)?*

*35.42/64.58*

***Comment:*** *The participants almost unanimously agree on the necessary contribution of neurosurgeons in the design of novel neuro-oncological treatment protocols.*

*Similarly, consensus was almost unanimous on the need for more multicenter randomized trials in this field, although the limits and challenges of these trial have already been addressed. In this context, it is well known that the expertise of the center and the surgeons may result in a significant bias. As an example, maximal safe resection may be differently reached and evaluated in distinct hands and settings. Thus, centralization in evaluation of presurgical conditions (e.g., MRI) and post-surgical results (e.g., pathological exam and postoperative MRI) among different neurosurgical centers may overcome these limitations in order to produce more homogeneous results.*

## Discussion

1. **Ethical considerations relating to clinical trials in pediatric neuro-oncology**

It is common for physicians, including pediatric neurosurgeons, to be involved in the design and conduct of pediatric neuro-oncology clinical trials. Whilst it is generally accepted that clinical trials must adhere to strict ethical requirements, it is also acknowledged that there are additional ethical considerations in children compared with adults. Although the legislation governing clinical trials varies between nations, typically children and adolescents under the age of 18 require the consent of their legal guardians (usually their parents) to participate in research studies. The process for obtaining consent is the same as for an adult research participant [102]. One characteristic of pediatric medicine is the triangular relationship of treating physicians, the children, and their parents. Parents of children with newly diagnosed brain tumors find themselves in an extraordinary situation. In addition to the realization of what is actually happening to their child and feeling the pressure to make the right decision about their child’s treatment plan, they may face many other stressors that may compromise their ability to make carefully considered choices, particularly in the emergency situation, he question of participation in clinical research trials adds another layer of stress, as parents are asked to make decisions about research at a time when “usual decision-making patterns become strained and tensions among family members increase” [103], or as one patient put it, “in a world of pain” [104]. This harbors additional potential for tension in the already conflict-laden triadic relationship between physicians, pediatric patients, and their parents.

Enrolment into clinical trials may invoke a conflict between utilitarian and individual benefit. While it is reasonable to assume that a high rate of child participation in clinical trials might be associated with significant advances for future treatments, the benefit to the individual, for example, in the option of biopsy or no biopsy is less tangible. The objectives of research and individual therapy need to be carefully balanced to ensure that ethical boundaries and distinctions between clinical care and research are not blurred.

The parents themselves, who are usually the surrogate decision-makers and provide informed permission for the enrollment of their child in a clinical trial, are actually not the study subjects. Parental decision-making is also driven by psychological reasons and enrolling their child in clinical trials may result in benefits for the parents (like hope) but not for the child. As a highly vulnerable group of patients [105], children must be protected from becoming a means to an end for the treatment team or parents, but must always remain an end in themselves, thus fulfilling Kant’s self-purpose formula (the categorical imperative is, firstly, the commandment to “never treat all others merely as means, but always at the same time as ends in themselves,” as it says in the Groundwork for the Metaphysics of Morals). Therefore, the best interest of the child [106] must always be the primary consideration in clinical treatment planning and clinical trial design. The “do no harm”-ethical principle [107] must be delicately balanced with the autonomy of the parents. But also here, the child’s best interest is paramount, and parental autonomy is tied to that.

With regard to parental autonomy, it is important to recognize that the distress of patients or their caregivers in oncology care may reduce their ability or competence to make autonomous decisions. Robertson et al. reported that the most common difficult medical decision identified by parents of children diagnosed with cancer was enrollment in a clinical trial [104]. Additionally, they found that parents of children entering clinical trials were exposed to a “flood of information but lack of understanding.” Adolescents in particular may have trouble in understanding and recalling treatment information [104]. The resulting parental and adolescents’ discomfort fed the notion that most of the affected parents do not really feel an urgent need to make decisions in this situation, preferring a paternalistic relationship with their child’s clinicians. Apart from the fact that paternalism as an outdated concept is not an option in current clinical practice, there is evidence that active involvement of parents and adolescents in cancer treatment decisions improves decision satisfaction and reduces the risk of decisional conflict and regret [108]. Therefore, current guidelines in pediatric oncology recommend that physicians provide developmentally relevant medical information to the child/adolescent and their parents so that the family can gain a better understanding of the disease and assume a more autonomous role to actively participate in medical decision-making processes, including clinical trial participation [109]. This concept of shared decision-making (SDM) [110] as good medical practice should be the basis for involving parents and pediatric patients in clinical trials.

As with other clinical decision-making processes, information about clinical trials should clearly state the intent, background, and design of the study. The treatment team must ensure that the decision-makers are well-informed and understand all aspects of the study [111]. Clinicians should be cognizant of a potential misunderstanding of the concept of clinical trials, i.e., parents may “not understand that the defining purpose of clinical research is to produce generalizable knowledge, regardless of whether the subjects enrolled… may potentially benefit from the intervention under study or other aspects of the clinical trial” [112]. This is ethically problematic for the conduct of clinical research, as it calls into question and undermines the validity of subjects’ informed consent [113].

Other misunderstandings that might compromise the validity of research trial consent include unrealistic optimism and other unrealistic therapeutic beliefs, such as therapeutic misestimation and therapeutic optimism [113]. Therapeutic misestimation may occur when subjects overestimate the benefits that a study can grant them or when they underestimate the potential risks associated with a particular study.

Therapeutic optimism describes a participant’s belief they will benefit from the study treatment, despite the express goal of RCTs to test unknown aspects of interventions.

Consequently, the doctors providing information should address these potential misunderstandings directly from the beginning to the parents and adolescents. For example, it should be clearly explained what a randomized or phase I trial means, even if there is a possibility that parents will be reluctant to participate [113].

Given these potential ethical pitfalls of research trial enrollment, particular emphasis should be placed on the deliberation phase rather than the decision phase of SDM. In this context, clinicians should also be aware that parental distress plays an important role at each stage of the deliberation and decision process [104]. Parents reported feeling “emotional” or “shutting down” due to the large amounts of information [104]. They felt unable to use the information they were given to make a decision and unable to comprehend the large amount of information in the time available to make a decision. Feeling pressured to make a decision again contributed to distress, which led to parents feeling unable to participate in the decision-making process [104]. Therefore, psychological support in every phase of SDM is necessary and should be offered as standard support to enable parents and adolescents to cope with their distress when making decisions about medical trials. In this respect, both parents and adolescents may benefit from improved quality of written information, including online, and QPLs (structured lists of common questions) [114] to facilitate communication.

When conducting medical trials with children and adolescents, the involved physicians have to be aware of the emerging and future autonomy of their patients. The child should be involved in decision-making in a manner that is adapted to the child’s or adolescent’s current level of intellectual development. Furthermore, every effort should be made to ensure that the child grows into an autonomous individual in the future, corresponding to the child’s right to an open future [115]. In pediatric medicine, there is also the option and in some cases the requirement of obtaining assent from an adolescent patient. “Assent refers to a child’s agreement or approval to participate in the care agreed upon by the parent” [116, 117]. However, the age at which children are capable of providing assent varies and there is no general consensus about this aspect [113]. There is the notion that “assent holds more weight in research deliberations than in routine clinical care, and a child’s dissent (i.e., refusal to assent) ought to be respected for nearly all research, except where research participation offers prospect of direct benefit, and is unavailable outside of the research context” [113]. To begin with, clinicians have to be trained to fulfill their ethical obligations to provide appropriate information to their pediatric patients, while respecting their developing autonomy [118, 119].

Clinical trials in pediatrics are needed to provide evidence-based treatment and establish a gold standard by which to evaluate future innovations are randomized-controlled trials (RCTs) and prospective comparative studies [120]. The respective study designs require particular ethical vigilance in pediatric medical subspecialties. One reason for this is the not uncontroversial concept of “equipoise” (i.e., “the ‘state of professional uncertainty about [the] relative therapeutic merits’ of the two treatments being studies” [121])*.* In a usual setting, physicians recommend the currently best available treatment option. Therefore, parents may feel insecure if treatment decisions in an obviously medically life-threatening situation are made randomly, rather than by their treating doctor in whom they have confidence. Further, ignoring personal preferences of both, the parents and the physicians, is always difficult and challenges the concept of equipoise. Further deliberations by ethical experts are needed in order to develop an ethical framework that could help navigate these obstacles. For now, the consensus is that the initial conditions, i.e., the equipoise, must be closely and independently re-evaluated during the course of the study, in order to recognize a potentially advantageous treatment option at an early stage. Therefore, RCTs require Data Safety Monitoring Committees to perform interim data analyses to ensure that equipoise is maintained or that one treatment option is superior to the other, which is the endpoint of the clinical trial [113].

2. **Unanswered questions and future perspectives**

In the molecular era, obtaining tumor material is the cornerstone of treatment. Thus, in the future, we can expect expanding indications to perform surgical biopsy aiming to define the molecular signature of the tumor, unless less invasive options are available. At this moment, *BRAF* and *H3K27M* mutations can be identified from CSF but it is not the standard and liquid biopsy is yet to come.

Knowing the molecular makeup of a tumor may provide a more accurate diagnosis and prognosis, more accurate information to the parents, and better stratification for adapted treatment intensity. That has become standard of care in many pediatric neuro-oncological centers if tissue samples are provided. In some cases, indication for biopsy may have changed in the context of finding potential druggable targets. A clear example is given by optic pathway gliomas. In the past, biopsy was reserved to unusual cases or to cases enrolled in a clinical trial [122] while nowadays tissue sampling is indicated for patients with non-NF-1 associated optic pathway gliomas and for those with NF-1 who demonstrate relevant mass effect or obvious progression of the disease or may have particularly atypical imaging features [123–126].

However, the progress of knowledge in terms of clinical implications and targeted or tailored treatments is still far behind what we know in terms of biology. The mere presence of a pharmacological or molecular target in a tumor does not necessarily indicate the use of a targeted therapy. Surgery still plays a leading role in the management of pediatric CNS tumors. The choice to start treatment with a targeted therapy must always be the result of a multidisciplinary discussion involving neurosurgeons, oncologists, pathologists, and radiotherapists in order to optimize the treatment to the patient’s characteristics.

In this context, we should highlight the importance of target validation, that is essential to differentiate target alterations driving the oncogenesis (drivers) from alterations that are not directly involved in the oncogenetic pathway (passengers).

After this premise, answers to questions of the consensus conference were difficult to dichotomize but the panel stimulated the discussion on the following issues.

- *Question 1: What is the current role for targeted therapies as neoadjuvant treatments before pediatric brain tumor removal?*
  The neoadjuvant role of targeted therapies is yet not defined, and many different tumor entities are to be considered. Furthermore, the molecular heterogeneity of even a single family of tumor entity, such as gliomas, will complicate the search for a single treatment approach that works for all cases.
  It is hoped that targeted therapies will be used to make unresectable tumor easier to resect. Unfortunately, there are no data to support such a hypothesis so far. Additionally, data are also lacking for when to stop targeted therapy and/or switch to surgery. The risk of developing mutations, thus escaping the action of the drug, should be evaluated in malignant tumors as this could be a reason for missing a window of opportunity for surgical treatment.
  Thus, it is too early to answer the above raised question. Until today, individual multidisciplinary decision-making will remain the precondition for making therapeutic decisions at the given time point. Future trials with strict protocols are required to define these points also within a multidisciplinary platform.

b. *Question 2: What are the benefits, cost/efficiency, and long-term side effects of targeted therapies in the treatment of pediatric brain tumors?*
  Currently, there are more questions than answers in respect to the risk/benefit profile of targetted therapies. Cost is also a factor limiting the availability of these drugs on a broader basis, particularly in public health systems.
  When dealing with benign tumors, the main concerns on targeted therapy are the chronic effects. As the long-term effects of these agents remain unknown, long-term data are required to provide appropriate and balanced counseling.
  Especially when dealing with malignant tumors concerns exist regarding the risk of new mutations leading to resistance or escape mechanism and fast tumor progressions, thus potentially missing the window for effective surgical treatment. Understanding resistance mechanisms with repeated tissue investigation may also identify other biologic targets that could be exploited for other treatment strategies in the due course. In this context, the safety and efficacy, as well as the durability, of these drugs in combination with other agents such as chemotherapy as well as other treatment strategies such as surgery and radiation therapy also require further study.
  On these grounds, future molecular studies should hopefully identify biomarkers potentially aiming to risk stratify patients, predict response and understand mechanisms of resistance. While progression on targeted therapy occurs in some patients, earlier detection of resistance would allow for change in management and potentially reduce morbidity.

c. *Question 3: Based on contemporary data, at which stage and in which pathologies do targeted therapies play a significant role*
  This consensus report offers a contemporary perspective on the state of the art of available targeted therapies in pediatric brain tumors. Most data are currently coming from study of LGG. In this context, trials have demonstrated superiority of targeted therapy compared to chemotherapy both in randomized trials and compared to historical cohorts within a limited time frame of follow-up in the subset of *BRAF V600E* mutant pediatric LGG. Trials investigating targeted therapy versus chemotherapy are ongoing in other LGG cohorts.
  The same drugs combination, BRAFi and MEKi, may be effective in HGG relapsed, progressed, or failed to respond to first-line therapy. Similar conclusions on efficiency may be drawn for mTORi in TSC and selumetinib in plexiform neurofibromas.
  For other tumors, the picture is still obscure due the lack of data or even the lack of druggable targets.
  Regarding targeted therapies with a proven efficacy, as BRAFi and MEKi in LGG, future studies should investigate the role of upfront treatment alone, or in combination with other treatments, such as surgery, chemotherapy, or radiotherapy, although targeted therapies usually aim to delay or avoid radiotherapy. To date, most trials have been designed to use the targeted agent for 18–24 months and many patients recur after this; thus, the optimal length of treatment to ensure a durable response is unclear. At recurrence following targeted therapy, patients may respond to the same agent (or class of agent), but whether the same degree of response can be achieved at later stages remains unknown. Additionally, the effect of these drugs on the natural history of the tumor should be studied, as we know that many LGG will ultimately undergo tumor senescence [96].

In conclusion, targeted treatment may not always be the best option even when a potential target is identified, and it should be remembered that surgery can still be the best option if the risk is well balanced in the individual case.

## Conclusions

This consensus report offers a contemporary perspective on advances in the field of targeted therapies in pediatric brain tumors from a multidisciplinary meeting of international experts. Whilst progress has clearly been made, this is an evolving field in which the place of targeted therapies and their role alongside neurosurgery needs to be better defined.

## Data Availability

No datasets were generated or analysed during the current study.
